# Occupational risk factors associated with respiratory symptoms among tannery workers in Mojo town, Southeast Ethiopia, 2018: a comparative cross-sectional study

**DOI:** 10.1186/s40248-019-0188-1

**Published:** 2019-08-05

**Authors:** Innawu Dalju, Awrajaw Dessie, Laekemariame Bogale, Tesfaye Hambisa Mekonnen

**Affiliations:** 1 Oromia Labour and Social Affairs Bureau, Oromia Regional State, Addis Ababa, Ethiopia; 2 Department of Environmental and Occupational Health and Safety, Institute of Public Health, University of Gondar, Gondar, Ethiopia

**Keywords:** Respiratory symptoms, Comparative cross-sectional, Tannery factories, Ethiopia

## Abstract

**Introduction:**

Work-related respiratory diseases (WRDs) account for 10–20% of all chronic respiratory illnesses affecting hundreds of millions of people of all ages. Tannery industries are often associated with hazardous working conditions favourable for respiratory conditions. However, information about the prevalence and occupational factors that predispose to respiratory symptoms is meagre in Ethiopia. This study aimed to investigate the magnitude and risk factors associated with work-related respiratory symptoms among tannery industry workers in Mojo town, Ethiopia.

**Methods:**

A comparative cross-sectional study was conducted from March to May 2018. A total of 602 (299 exposed to tannery factories) and 303 unexposed (civil servants) were included using the stratified sampling method. The British Medical Research Council (BMRC) questionnaire was pretested and interviewer-administered for data collection. A binary logistic regression analysis was performed to identify the factors associated with respiratory symptoms. The significance of associations was ascertained at a < 0.05 *p* and adjusted odds ratio with a 95% CI was computed to evaluate the strength of associations.

**Results:**

The prevalence of respiratory symptoms among exposed workers was 27.1% [95% CI (21.7, 32.1)] and 8.3% [95% CI (5.3, 11.6)] among unexposed workers in the previous 12 months and the prevalence was significantly different (X^2^ = 36.82; *p* < 0. 00001). The odds of developing respiratory symptoms was 3.37 times higher among tannery workers than unexposed workers [AOR: 3.37; 95% CI (1.71, 6.46)]. Female sex [AOR:1.80; 95% CI (1.24, 3.34)], temporarily workers [AOR = 3.43; 95% CI (2.63, 7.95)], working in a poorly ventilated working unit [AOR = 1.88; 95% CI (1.22, 3.98)], absence of occupational health and safety training [AOR = 2.37; 95% CI (1.14, 4.92)], and not using personal protective equipment [AOR = 2.30; 95% CI (1.25, 3.46)] were significant factors.

**Conclusion:**

The prevalence of respiratory symptoms was higher among exposed workers compared to unexposed ones. Sex, employment status, ventilation of working units, absence of occupational health and safety training, and not using personal protective equipment were the factors associated with occupational-related respiratory symptoms. Strategies targeting health and safety training, creating awareness on the use of personal protective equipment, and improving workplace conditions, like the provision of adequate ventilation are useful means for assuaging the condition.

## Background

The World Health Organization (WHO) 2017 report indicates that non-communicable diseases (NCD) are the leading causes of death accounting for 70% of all deaths worldwide [1]. About 63% (36 million) of these were due to respiratory diseases (contributing to 4.2 million deaths) and other non-communicable diseases. Respiratory problems always appear after different respiratory symptoms [2].

Respiratory disorders from occupational sources are the major public health problems, accounting for 30% of all registered work-related diseases and 10–20% of deaths worldwide [3]. Owing to exposure to occupational airborne particulate matters, an estimated 386,000 deaths and nearly 6.6 million disability adjusted life years (DALYs) occur among workers [4]. In low and middle-income countries, including Africa, occupational respiratory symptoms and diseases are a huge burden due to the prevailing poor working conditions and environments and the use of obsolete machines with a potential to generate excessive dusty hazards [2].

Exposure to dust and chemicals released from the tannery industry is one of the major causes of respiratory disorder profoundly affecting health of working population [5–7]. Chemicals used in tannery industries for the preservation of raw hides, like chromium salts, have the potential to bind with skin proteins. This could produce complex antigens and lead to hypersensitivity and subsequently to the development of respiratory diseases [8]. Tannery workers are thus potentially exposed to harmful agents rendering them vulnerable to health problems, especially of the respiratory tracts and skin.

Dust is produced in a variety of tanning operations. Chemical dust can be produced during the loading of hide-processing drums. Leather dust is produced during mechanical operations. Buffing is the major source of dust. The dust in tanneries may be impregnated with chemicals, as well as fragments of hair, mould, and excrement [6]. Exposure to chemicals and organic solvents, such as chromate and bichromate salts, aniline, butyl acetate, ethanol, benzene, toluene, sulfuric acid, and ammonium hydrogen sulfide used in the tannery industry also leads to respiratory disorders [9]. The getting in of chromium into the body through inhalation, ingestion, and by direct cutaneous contact increases the risk of dermatitis, ulcers, and perforation of the nasal septum leading to respiratory illnesses as well as increased lung and nasal cancers [10, 11]. Chromium specific health hazards, like the carcinoma of the larynx, lung parenchyma, and para-nasal sinuses have also been reported [10, 12].

Tannery industries are economic backbones in many countries, generating elevated employment opportunities. In African countries, like Ethiopia, tannery industries have been rapidly growing since recently in order to raise the capacity to export leather products [13]. Ethiopia is one of the richest countries in Africa in livestock resources. Currently, there are 33 tannery industries in operation and more than ten thousand workers are employed in the sector [14, 15].

Together with the advent of the sector expansion, however, workers employed in the industry are at a high risk of experiencing respiratory problems due to the increasing dust hazards it often generates; but the degree of the problem is not well known and there has been a scarcity of data showing these kinds of health issues and its associated factors in Ethiopia. Therefore, this study was conducted to assess a self-reported occupational- related respiratory symptoms and associated factors among tannery factory workers in Mojo town, Southeast Ethiopia.

## Methods

### Study design, setting, and population

A comparative cross-sectional study was conducted from March to April 2018 among tannery workers in Mojo town.

The source population was all workers in Mojo town tannery industries in pre-tanning, tanning, and finishing departments. During the data collection period, a total of 12 tannery industries were found in Mojo town employing more than 7,520 workers. Those who worked for at least a year in the industries were included in the study. Workers with previous exposure to other occupational dust, such as silica, and coal dust and had history of smoking were excluded. Furthermore, workers who had history of asthma or chronic obstructive pulmonary disease (COPD) before joining the industries and who were past and current cigarette smokers were also excluded from the study.

Of the activities carried out in working units, pre-tanning involves the preservation and preparation of hides. In this process, workers are at risk of an exposure to hazardous chemicals [16]. In the tanning production, a processed hide is converted into tanned leather by stabilizing the collagen structure by chromium sulphate, the most widely used tanning agent. In the finishing or post tanning department, the final product or leather is treated with chemicals and prepared for packaging [16].

The source populations for the unexposed group was both the general administration staff of the tannery factories and external workers in the public administration (civil servants) in Mojo town, with at least a year of service. Those who had history of current and past smoking, asthma or COPD were excluded from the unexposed group.

### Sample size determination

The sample size was determined using the double population proportion formula. The assumptions considered to calculate the sample size were a 26.6% proportion of respiratory symptoms among the exposed group [17] and 11.5% among the unexposed group (11.5%) [17], a 95% confidence interval, 80% power, 5% margin of error, 1:1 ratio of exposed to unexposed group, and a 10% non-response rate, which yielded a sample of 602.

### Sampling procedures

To recruit participants of the exposed group, we first randomly selected six tannery factories from among the twelve tannery factories found in Mojo town, Southeast Ethiopia. Next, the desired participants were selected using the stratified sampling technique, assuming that the workers in different working sections would exhibit different levels of exposure to chemicals and dusts generated from the working departments. Finally, the participants were selected proportionally from each strata namely, pre-tanning, tanning and finishing or post-tanning stratas.

### Measurement of variables

Respiratory symptoms, like cough, phlegm, wheezing, dyspnea, chest pain, chest tightness and breathlessness that lasts for three months among workers determined the presence of respiratory symptoms, the primary outcome variable of the study, in the past one year.

The ventilation condition of working units was reported as good if the unit is furnished with functional mechanical ventilation systems (ventilator, local exhaust ventilation system) and natural ventilation systems (doors, windows, and any other openings). Poor ventilation, if the unit lacks functional mechanical and natural ventilation systems and if the air flow is obstructed by adjacent buildings and poor layout of the unit.

### Data collection procedures

The modified British Medical Research Council (BMRC) questionnaire was used for data collection [18]. This instrument has been widely used in the literature [19, 20]. A recent study conducted in Ethiopia also employed the instrument which was customized to the local culture and conditions [19]. Overall, the detailed questions designed consisted of four parts, viz., socio-demographic, behavioral, occupational as well as environmental factors, and respiratory symptoms. Face-to-face interviewing and observation of the working units were performed to collect data. A two day was given to data collectors and supervisors on procedures, techniques, and ways of collecting data. Besides, a clear introduction was provided about the purpose and objectives of the study to the respondents before data collection. In addition, continuous and strict supervision and on the spot checking was carried out during the process.

### Data processing and analysis

The data were checked, coded, and entered in to the epidemiological information package (EPi-info) version 7.2.0.1 and exported to statistical package for social sciences (SPSS) version 20 for further analysis. For most variables, data were presented as frequencies and percentages. A bivariate logistic regression analysis was performed primarily to select variables for the final model on the basis of a < 0.2 *p*. A multivariable binary logistic regression analysis was employed to control the possible effects of confounders. Finally, variables which showed significant associations were identified on the basis of the adjusted odds ratios (AOR) with a 95% CI and p- ≤0.05.

## Results

### Socio demographic characteristics

A total of 602 participants (299 exposed and 303 unexposed) with a response rate of 98% fully responded to the interview questions. The mean ages of the participants in the exposed and unexposed groups were 27 (SD ± 5.23) and 32 (SD ± 5.00) years, respectively. The overall mean age was 29 (±5.3) years. The majority of the respondents, 74.3% (*n* = 223) and 86.8% (*n* = 263), were male in the exposed and unexposed groups, respectively. Out of the total respondents, 53.2% (*n* = 159) were exposed and 44.2% (*n* = 134) unexposed and served for 1–4 and 5–10 years in their respective workplaces, respectively. The majority of the participants had attended secondary schools and above (Table 1).

**Table 1:** Socio-demographic characteristics of tannery and Civil servant workers, Mojo town, southwest Ethiopia, 2018

Variables (*N* = 602)	Exposed (*n* = 299) Frequency (%)	Unexposed (*n* = 303) Frequency (%)	Chi-square Test, *P*
Sex			0.0002
Female	76(25.4)	40(13.2)	
Male	223(74.6)	263(86.8)	
Age (years)			< 0.00001
18–25	109(36.5)	36(11.9)	
26–34	131(43.8)	191(63)	
> 35	59(19.7)	76(25)	
Marital Status			0.04
Single	121(40.5)	121(39.9)	
Married	157(52.5)	174(57.4)	
Divorced/Widowed	21(7.0)	8(2.6)	
Work experience			< 0.00001
1–4	159(53.2)	99(32.1)	
5–10	57(19.1)	134(44.2)	
10+	83(27.8)	70(23.1)	
Ethnicity			< 0.00001
Oromo	181(60.3)	249(82.2)	
Amhara	72(10.0)	48(15.8)	
Others	46(15.7)	6(2)	
Religion			0.002
Orthodox	161(53.8)	170(56.1)	
Protestant	90(30)	59(19.5)	
Muslim	48(16)	74(24.4)	
Educational Status			< 0.00001
Primary school	105(35)	1(0.3)	
Secondary school	124(41.5)	7(2.3)	
Diploma and above	70(23.4)	295(97.3)	
Monthly salary in ETB			< 0.00001
< 1500	150(50.2)	21(6.9)	
> 1500	149(49.8)	282(93.)	

Keys: *ETB* Ethiopian birr (national currency) (1 $ USA = 28 ETB)

### Workplace characteristics

According to this study, 65.6% (*n* = 196) of the exposed and 94.1% (*n* = 285) of the unexposed said that they had no training on occupational health and safety. Of the tannery workers, 75.9% (*n* = 227) reported exposure to different chemicals, while just two (0.7%) of the civil servant workers reported a chemical exposure at the workplace. This showed that about 68.9% (*n* = 206) of the participants among exposed group reported that they were exposed to leather dust, whereas 2% (*n* = 6) of the unexposed group reported they had exposure to leather dust in their workplace. Furthermore, about 65.2% (*n* = 195) of the exposed group and 2.6% (*n* = 8) of the unexposed group reported that they used personal protective equipment (Table 2).

**Table 2:** Workplace characteristics of the participants Mojo twon, Southeast Ethiopia, 2018

Variables (N = 602)		Exposed (n = 299) Frequency (%)	Unexposed (n = 303) Frequency (%)	Chi-square Test, *P*
OSH Training	No	196(65.6)	285(94.1)	< 0.00001
	Yes	103(34.4)	18(5.9)	
Chemical Exposure	No	72(24.1)	301(99.3)	< 0.00001
	Yes	227(75.9)	2(0.7)	
Leather dust exposure	No	93(31.1)	297(98.0)	< 0.00001
	Yes	206(68.9)	6(2.0)	
Ventilation	Poor	126(42.1)	292(96.4)	< 0.00001
	Good	173(57.9)	11(3.6)	
Home energy use	Polluted	210(70.2)	275(90.8)	< 0.00001
	Clean	89(29.8)	28(9.2)	
Periodic medical examination	No	192(64.2)	296(97.7)	< 0.00001
	Yes	107(35.8)	7(2.3)	
PPE use	No	104 (34.8)	295(97.4)	< 0.00001
	Yes	195(65.2)	8(2.6)	
Previous exposure to dust working environment	No	231(77.3)	302(99.7)	< 0.00001
	Yes	68(22.7)	1(0.3)	

Keys: *N* number (total), *n* number, *OHS* Occupational health and safety, *PPE* personal protective equipment

### Behavioral characteristics

In this study, 22.4% (*n* = 67) of the exposed and 31.7% (*n* = 96) of the unexposed group indicated that they drank alcohol twice a week; 26.1% (*n* = 78) of the exposed and 26.7% (*n* = 81) of the unexposed respondents reported that they performed physical exercise more than twice a week, and 9.4% (*n* = 28) of the exposed and 9.3% (*n* = 19) of the unexposed reported they were khat chewers.

### Prevalence of respiratory symptoms

**Fig. 1:**
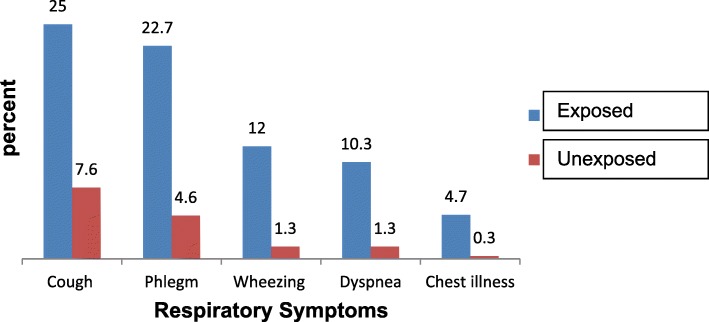
Prevalence of respiratory symptoms among tannery and civil servant workers, Mojo town, Southwest Ethiopia, 2018 (*N* = 602)

This study demonstrated that the prevalence of self-reported respiratory symptoms was 27.1% (n = 81); [95%CI (21.7, 32.1)] and 8.3% (*n* = 25); [95%CI (5.3, 11.6)] among the exposed and unexposed groups, respectively. The prevalence was significantly higher among the exposed group compared to unexposed (X^2^ = 36.82; *p*< 0.00001). The perceived symptoms noted among the tannery industry workers (exposed group) were cough 25%, phlegm 22.7%, wheezing 12%, dyspnea 10.3, and chest illness 4.7% (Fig. 1).

### Associated factors of respiratory symptoms

Two models were fitted in this study, one large model for the exposed and unexposed groups together and the second for the exposed group alone. Sex, educational status, work experience, age, and exposure status were entered in the former model. After adjusting confounding, like sex, age, work experience, and educational status, exposure status was significantly associated with respiratory symptoms. The odds of respiratory symptoms was 3.37 higher among the exposed group than the unexposed group [AOR:1.80; 95% CI (1.24, 3.34)]. Sex and educational status were also associated with respiratory symptoms in the larger model (Table 3).

**Table 3:** Multivariable analysis of factors associated with respiratory symptoms among exposed and unexposed groups, Mojo town, Southeast Ethiopia, 2018 (N = 602)

Variables	Respiratory symptoms		COR (95% CI)	AOR (95% CI)
	Yes	No		
Exposure status				
Exposed	81	218	4.13 (2.55, 6.69)^a^	3.37 (1.71, 6.46)^b^
Unexposed	25	278	1	1
Sex				
Female	34	88	2.18 (1.37, 3.50)^a^	1.80 (1.24, 3.34)^b^
Male	72	408	1	1
Age (years)				
18–24	31	113	1.30 (0.70, 2.43)	1.39 (0.62, 3.08)
25–34	55	288	0.90 (0.52, 1.59)	1.12 (0.58, 2.12)
35+	20	95	1	1
Work experience (years)				
1–5	37	221	1	1
6–10	36	155	1.38 (0.83, 2.29)	0.60 (0.32, 1.15)
> 10	33	120	1.64 (1.97, 2.76)^a^	1.42 (1.19, 2.18)^b^
Educational status				
Primary (grade 1–8)	30	76	2.92 (1.88, 5.39)^a^	1.52 (1.27, 4.79)^b^
Secondary (9–12)	33	101	2.42 (1.56, 4.31)^a^	1.24 (1.16, 3.87)^b^
Diploma and above	43	319	1	1

Keys: ^a^significant in a bivariate analysis; ^b^Significant in a multivariable analysis

In the second model, sex, educational status, experience, age, employment status, ventilation, chemical exposure, OHS training, PPE use were entered. The output of the multivariate binary logistic regression showed that sex, employment status, ventilation, OHS training, and PPE use were significantly associated with respiratory symptoms (Table 4).

**Table 4:** Multivariable analysis of factors associated with respiratory symptoms among exposed groups (tannery factory workers), Mojo town, Southeast Ethiopia, 2018

Variables	Respiratory symptoms		COR (95% CI)	AOR (95% CI)
(n = 299)	Yes	No		
Sex				
Female	31	50	2.03 (1.23, 3.67)^a^	1.64 (1.17, 3.51)^b^
Male	50	167	1	1
Educational status				
Primary (Grade 1–8)	30	74	1.09 (0.55, 2.14)	1.01 (0.46, 1.89)
Secondary (Grade 9–12)	32	93	0.92 (0.48, 1.79)	0.67 (0.37, 1.76)
Diploma & above	19	51	1	1
Work Experience (years)				
1–5	41	112	1	1
6–10	12	61	0.54 (0.26, 1.10)	0.76 (0.42, 2.13)
> 10	28	45	1.70 (0.94, 3.07)	1.42 (0.86, 2.86)
Age (years)				
18–24	28	81	1.12 (0.64, 1.95)	1.10 (0.28, 1.88)
25–34	42	109	1.14 (0.50, 2.58)	1.08 (0.34, 1.76)
35+	11	28	1	1
Employment status				
Permanent	54	199	1	1
Temporary	27	19	5.24 (2.71, 10.13)^a^	3.43 (2.63, 7.95)^b^
Ventilation				
Poor	47	80	2.38 (1.42, 4.01)^a^	1.88 (1.22, 3.98)^b^
Good	34	138	1	1
Chemical exposure				
No	21	51	1	1
Yes	60	167	0.87 (0.48, 1.57)	0.65 (0.46, 1.53)
OHS training				
No	64	130	2.55 (1.40, 4.64)^a^	2.37 (1.14, 4.92)^b^
Yes	17	88	1	1
PPE use				
No	42	64	2.59 (1.53, 4.38)^a^	2.30 (1.25, 3.46)^b^
Yes	39	154	1	1

Keys: ^a^significant in a bivariate analysis; ^b^Significant in a multivariable analysis

Sex was associated with respiratory symptoms among tannery workers. Accordingly, compared to males, female workers had 1.80 higher odds of developing respiratory symptoms [AOR:1.80; 95% CI (1.24, 3.34)]. Employment status was also found to be significantly associated with the problem. The odds of developing respiratory symptoms were 3.43 times higher among temporarily employee workers than their counterpart permanent employees [AOR:3.43; 95% CI (2.63, 7.95)].

Workers in poorly ventilated working units had 1.88 times higher odds of developing respiratory symptoms compared to their counterparts [AOR:1.88; 95% CI (1.22, 3.98)]. Furthermore, the odds of developing respiratory symptoms increased 2.37 times among workers who received no occupational health and safety training than workers who had the training [AOR: 2.37; 95% CI (1.14, 4.92)]. This study also revealed that workers who did not use personal protective equipment had 2.30 times more odds of developing respiratory symptoms compared to those who used the equipment [AOR: 2.30; 95% CI (1.25, 3.46)].

## Discussion

This is a workplace-based comparative cross-sectional study conducted on tannery factory and civil servant workers in Mojo town, Southeast Ethiopia. The prevalence of respiratory symptoms was 27.1 and 8.3% among exposed and unexposed groups, respectively. The findings revealed that the prevalence of respiratory symptoms among the exposed was higher compared to the unexposed. The difference might be due to the fact that tannery industry workers were more likely to be exposed to various workplace hazards associated with tannery activities, like chemical hazards, including chromium salt, acids, dyes, and leather dusts, and biological hazards from raw hides that might exacerbate respiratory symptoms. Differences in prevalence between exposed and unexposed groups were also observed in studies conducted in Kenya [1], Egypt [21, 22], Pakistan [23], India [24, 25], and Turkey [6]. Findings of the workplace observations also showed that chemicals used in production processes which are usually improperly handled might lead to the occurrences of respiratory disorders among tannery employees. Furthermore, workers who usually handled the chemicals were not those who were not authorized to do and the majority do not have any training and orientation on how to manage and use the chemicals. In addition, chemical safety data sheets were not accessible to all chemical handlers at the workplaces. During our observation, we also noted that there were poor housekeeping practices. The necessary local exhaust and general ventilations were not installed at the required locations, and combinations of all these hazards would be more likely to worsen the developments of several respiratory symptoms among tannery workers.

Our investigation showed that the prevalence of respiratory symptoms among the exposed groups was 27.1% (95% CI: 21.7, 32.1). This result was relatively comparable to those of studies in Karachi, Pakistan (25%) [17] and India (23%) [26]. The inherent nature of hazards associated with tanning procedures are often similar everywhere. However, the current finding was lower than those of studies conducted in Kenya (38.4%) [1], Sudan (38%) [27], Egypt (58.3%) [22], and Bangladesh (49.9%) [28]. The discrepancies could be due to the fact that there might be differences in assessment methods, sample size, injury and illness reporting procedures, and workplace health and safety cultures and practices across factories.

In the current study, we explored that sex was an important risk factor for work-related respiratory symptoms. The odds of developing respiratory symptoms were more likely manifested among female workers than males. This result was corroborated by another study [20]. The plausible suggestion might be that women are often responsible for multiple roles and responsibilities both at home and workplaces (home/work interface) that might increase their vulnerability to the symptoms. Further, the difference could be due to the common workplace gender segregation which is usually apparent in tannery industries. Female workers in tannery factories are usually assigned to the post-tanning or finishing departments where exposure to leather dust generated from finished goods or leather is highly likely. This usually occurs due to the assumption that post-tanning stages of the production process is regarded as light work compared to other sections, while it is major detrimental source of respiratory exposure to sources of dust. This was supported by the results of observations of workplace conditions. In accordance with our workplace observational results, the majority of the workers in the post-tanning (mainly mechanical operation areas) processes were female.

Employment status was found to be a significant factor of respiratory symptoms. Hence, temporary workers had higher odds of developing respiratory symptoms compared to permanent employees. The possible reason might be differences in benefit packages the industry provides to permanent and temporary workers. Thus, temporary workers have limited access to basic safety training and the use of personal protective devices. Furthermore, such groups of workers are usually forced to do more tasks for fear of losing their jobs or to stay on their jobs for more time [29, 30].

The ventilation of working units was significantly associated with respiratory symptoms. Higher odds of developing respiratory symptoms were observed among workers who worked in inadequately ventilated working units. This might be due to an increased accumulation of chemicals and dusts in poorly ventilated working environments which in turn result in respiratory symptoms. Furthermore, the effect of dusts and chemicals has a tendency to be more pronounced in poor ventilation [31]. Increased odds of respiratory symptoms were observed in other similar study [20] .

In this study, workers who did not use protective equipment were 2.30 times more likely to develop respiratory conditions than PPE users. This finding is similar to that of a study done in Pakistan [23]. The probable reason might be potential exposure to inhalable hazardous substances in workplaces would be prevented and reduced by using protective equipment, like respirators and masks. However, the current finding is inconsistent with that of a research in Ethiopia [32]. The contrasting result could be attributed to differences in the quality and availability of PPE, practices, and attitudes/believes towards using PPE and the measures in place to ensure compliance with PPE uses. This finding is supported by the results of workplace observations. Observations for the availability and practices of the use of PPE showed that the necessary respiratory protective devices, like protective respirators and/masks, were not in place. We noted that the very few available PPE were old, outdated, and not to the standard PPE supplied by recognized organizations. Furthermore, we confirmed that almost all of the tannery employees observed never used PPE. The workers usually raised several reasons for not using protective devices. Some workers indicated equipments were often uncomfortable and that they limit freedom, whereas others complain that company owners do not provide standardized personal protective equipment.

In the current study the absence of safety and health training was the other significant factor for work-related respiratory conditions. Workers who had no health and safety training were more likely to manifest respiratory symptoms compared to those who reported to have safety trainings. This was supported by a study done in Ethiopia [19]. Safety and health training might increase workers’ awareness, knowledge, and skills of the prevention and control of respiratory and other health conditions. Safety training might also positively influence employees' behavior in promoting workplace safety cultures and practices.

### Limitations of the study

The comparative cross-sectional method employed in this study is of great importance to evaluate whether exposure to the inherent nature of hazards in tannery working environments is significantly connected to occupational respiratory health disorders. The interview data collection method employed also increased the response rate that ensured confidence about the representativeness of the result. However, some limitations relating to the assessment method employed could not be ruled out. First, lung function and chromium exposure tests and air sampling for dust calculation were missing. Second, the data obtained for the evaluation of the prevalence of respiratory symptoms were based on employees' self-reports. Therefore, potential recall bias and under reporting might be anticipated. Third, the cross-sectional nature of the study makes it difficult to conclude the temporal-relationships between occupational-related factors and different respiratory symptoms developments. Furthermore, the study employed control group whose characteristics, such as age, sex, and educational status did not match with those of the exposed groups. This might reduce the statistical power of the study. However, the large sample size we employed and fitting a separate model to identify predictor factors for respiratory symptoms could ameliorate the effects of the comparison. Future studies may need to employ such strong study design as prospective/longitudinal and matched case-control, to confirm the plausibility of associations between work-related respiratory symptoms and occupational factors. Furthermore, this study used the BMRC questionnaire to measure respiratory symptoms because it was used widely in the literature and a recent study in the country (Ethiopia) employed this particular instrument that increased confidence and the customization of the tool to the country’s context. However, we recommend that future studies employ a new validated respiratory questionnaires to measure respiratory symptoms.

## Conclusion

The prevalence of respiratory symptoms was higher among exposed workers compared to unexposed ones. Sex, employment status, ventilation of working units, absence of occupational health and safety training, and not using personal protective equipment were the factors associated with occupational-related respiratory symptoms. Strategies targeting health and safety training, creating awareness on the use of personal protective equipment, and improving workplace conditions, like the provision of adequate ventilation are useful means for assuaging the condition.

The prevalence of respiratory symptoms was higher among exposed workers compared to unexposed ones. Sex, employment status, ventilation of working units, absence of occupational health and safety training, and not using personal protective equipment were the factors associated with occupational-related respiratory symptoms. Strategies targeting health and safety training, creating awareness on the use of personal protective equipment, and improving workplace conditions, like the provision of adequate ventilation are useful means for assuaging the condition.

## Data Availability

The datasets generated and analyzed in this study are not publicly available because personal information is contained in the data, but they are available from the correspondent author on reasonable request.

## Abbreviations

AO: adjusted odd ratio
CI: Confidence interval
COPD: Chronic Obstructive Pulmonary Disease
COR: Crude odd ratio
MPH: Masters of public health
MSC: Masters of science
PPE: Personal protective equipment
SPSS: statistical package for social science
